# Avoidable visits to the emergency department(ED) and their association with sex, age and race in a cohort of low socio-economic status patients on hemodialysis in the Bronx

**DOI:** 10.1371/journal.pone.0202697

**Published:** 2018-08-24

**Authors:** Ladan Golestaneh, Eran Bellin, Joel Neugarten, Yungtai Lo

**Affiliations:** 1 Department of Medicine, Renal Division, Albert Einstein College of Medicine, Montefiore Medical Center, Bronx, NY, United States of America; 2 Department of Epidemiology and Population Health, Albert Einstein College of Medicine, Montefiore Medical Center, Bronx, NY, United States of America; University of Liège, BELGIUM

## Abstract

**Background:**

In national samples drawn from the USRDS, female patients utilize the hospital ED and inpatient services at a higher rate than their male counterparts and have a higher rate of re-hospitalization. We wanted to explore the association of sex with avoidable ED visits made by a cohort of patients on hemodialysis in a mostly minority, lower socioeconomic status (SES), population in the Bronx to test the applicability of the USRDS findings.

**Methods:**

We used Montefiore’s clinical database to build a cohort of patients on hemodialysis with a first ED visit between 2013 and 2017. All ED visits after the index ED visit and those within one year prior to the index visit were recorded. None of the ED visits resulted in a hospitalization and were thus labeled “avoidable”. Bivariate analysis tested the association of demographic and clinical variables with sex. We used negative binomial regression to test the association of each variable with avoidable ED visit count. The multivariate model used negative binomial regression with avoidable ED visit count as outcome and sex as the exposure variable and included ancestral variables age and race. Potential mediators were added to the model to measure their effects on the association of sex with avoidable ED visits.

**Results:**

Four thousand six hundred and seventy three subjects on hemodialysis were identified as having at least one avoidable ED visit, in the period of 2013–2017 at one of four ED sites affiliated with Montefiore Medical Center in the Bronx. Over 5 years (2012–2017), the median number of ED visits made by the study sample was 4 (25–75% IQR: 2–8). Female patients on hemodialysis in our cohort were older, more commonly black, had lower SES scores, less commonly had commercial insurance and were less commonly married than their male counterparts. Female sex was not significantly associated with a higher rate of avoidable ED visits in the total cohort.(1.053(0.99–1.12) Female sex was significantly associated with outcome in non-Hispanic whites only and in those subjects younger than 44 years old.(IRR 1.30(1.06–1.69), 1.17(1.00–1.38) in non-Hispanic White and younger age group, respectively.) Marital status, SES and hemoglobin levels possibly mediated the association of sex and outcome in our population. (>25% change in the coefficient for sex with respect to outcome when variable added to the model).

**Conclusion:**

In this single center study of a lower-socioeconomic status, mostly minority dialysis population, the association of female sex with avoidable ED visits was not significant. These results suggest the association of sex with hospitalization outcomes, described by national datasets that determine quality indicators, are not consistent across different types of populations with some mediation possible by SES and marital status in poorer neighborhoods.

## Introduction

Patients on hemodialysis comprise 1% of the Medicare population but cost up to 9% of the Medicare budget.[1, 2] Hospitalizations account for up to 40% of this cost. Hospitalization costs neared 10 billion in 2013 and continue to rise.[3] Nationwide, thirty five percent of hospitalized patients on hemodialysis are readmitted within 30 days of discharge and patients on hemodialysis have a hospitalization rate of 1.7 per patient per year.[1, 2, 4] Thus, the high hospitalization rate in patients on hemodialysis accounts for part of their enormous cost to insurers. The Centers for Medicare Services (CMS) has made readmission rate a Quality Incentive Program (QIP) benchmark tied to dialysis facility reimbursements starting in 2017.[5–7] The QIP, however, does not adjust for case-mix or for socio-economic factors that contribute to the inability of certain vulnerable populations to meet quality benchmarks, opting instead to use benchmarks defined by large, claims based national datasets.[8–10] Psychosocial barriers to outpatient care, mental health issues related to the large burden of hemodialysis therapy and the lack of coordination between dialysis facilities, nephrologists and other service providers, contribute to avoidable ED visits, particularly in low socioeconomic class neighborhoods. [11–14] Community level healthcare disparities have been shown to contribute to poor outcomes in the ESRD population.[8–10] The reimbursement rates that are based on CMS quality indicators run the risk of not accounting for local socio-economonic contributors to outcomes, thus cutting funds to providers in underserved communities, and thus limiting the resources to areas that need them most.

The USRDS annual report from 2017 showed that female patients with ESRD have higher rates of hospitalizations than their male counterparts.[15] A recent study by Adams also showed that female patients had a higher rate of hospitalizations and 30 day readmissions across age groups and races, in a national sample of patients on hemodialysis.[16] Another national sample showed that female and younger patients on hemodialysis had higher incidence of ED visits, as did patients with Medicaid insurance, Black race, and lower SES.[11] However, none of these studies explored potential mediators of the association of female sex and hospitalization. The higher hospitalization rate in females may be attributable to sex and age-group differences in types of co-morbidities prior to dialysis initiation, barriers to nephrologist care and outpatient care coordination, and psychosocial burdens.[17, 18] In order to better understand the association of sex with hospitalization in patients on hemodialysis, described by national data, we wanted to study the association of female sex with ED visits in our local, poor, mostly minority population. Because most hospital admissions occur through the ED and greater than 50% of ED visits result in a hospitalization, a better understanding of ED visit patterns in patients on hemodialysis is warranted.[19]

We examined the association of sex with ED visit count over 5 years in a cohort of patients on hemodialysis in the Bronx, a low income, urban, largely minority population, and tested relevant clinical and socioeconomic variables for potential mediation. Our hypothesis states that community case-mix affects the association of sex with ED visit outcomes.

## Materials and methods

This study was approved by the institutional review board(IRB) of the Albert Einstein College of Medicine who waived the need for informed consent. We conducted a retrospective cohort study using clinical and administrative data from our Medical Center’s main database (Looking Glass™)[20, 21] over a period of 5 years. Looking Glass^TM^ Clinical Analytics (Streamline Health, Atlanta, Georgia) is a user-friendly interactive software application for the evaluation of health care quality, effectiveness, and efficiency. [20, 21]

### Identifying the cohort

**Fig 1:**
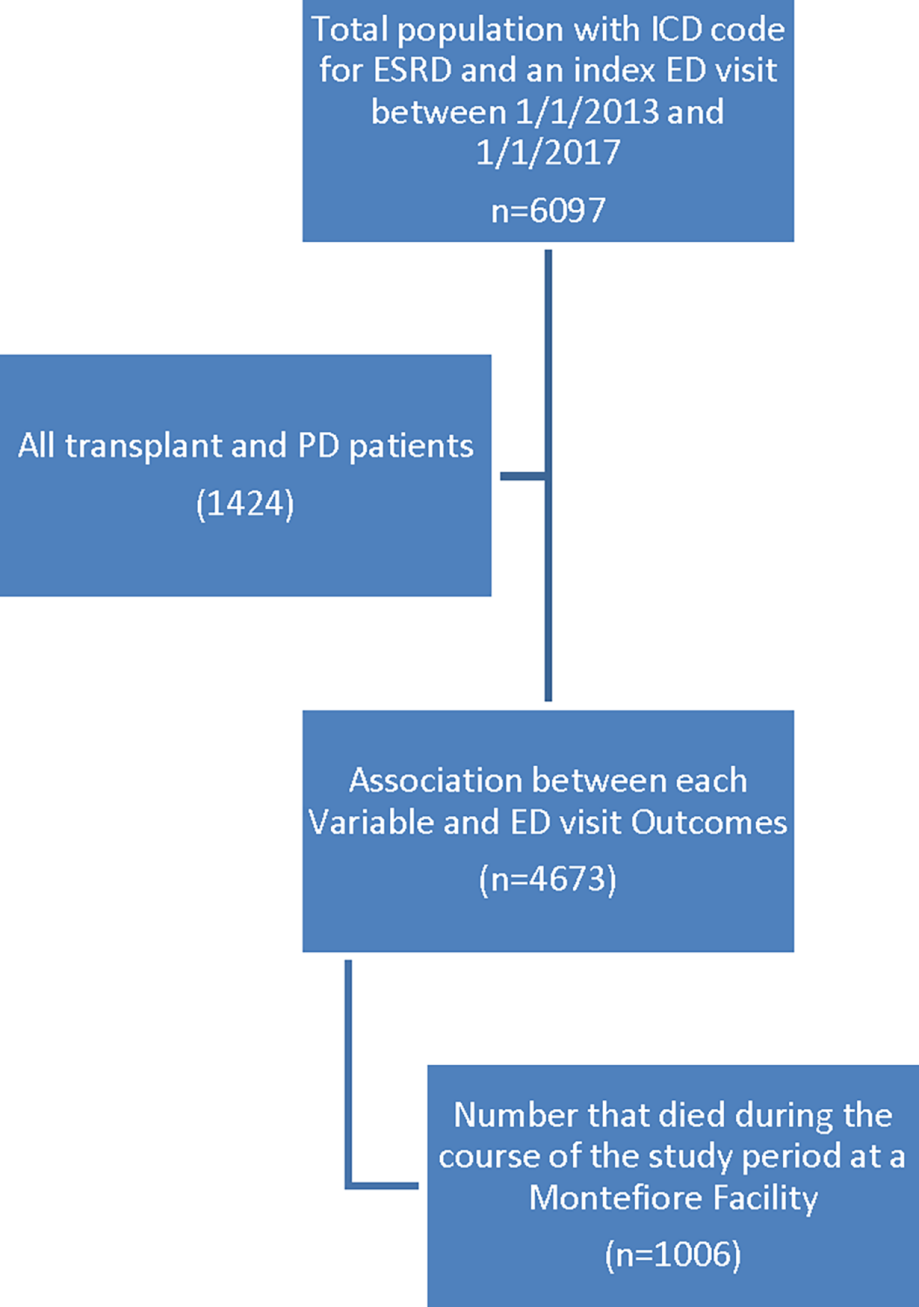
Identifying the cohort.

Using Looking Glass^TM^, we identified a cohort of patients on hemodialysis who had an index ED visit between 2013-2017(1/1/2013-1/1/2017), at any of three Montefiore Hospitals in the Bronx, NY. (S1 Data) We excluded transplant recipients and those on peritoneal dialysis. (Fig 1) We then gathered longitudinal data after the index ED visit on ED visits going forward until the end of the study period, and death events. We also recorded the number of ED visits made in the 365 days prior to the index ED visit (totaling 5 years of ED visit data). For this analysis we excluded all ED visits that led to hospital admissions, choosing instead to focus on ED visits that did not lead to an admission. We called this outcome variable: “avoidable ED visits” with the assumption that those ED visits that did not lead to an admission could have been avoided with better outpatient care coordination and care provision(Mostly through the dialysis facilities). We offset our analysis by duration of follow up, using date of death as the dropout date. Our outcome variable was the total count of ED visits over a 5 year period (Fig 1).

### Variables

After examining our causal model we gathered the following variables where data was available: 1-demographic (age, gender, race, ethnicity, socioeconomic status, primary language(English vs not-English, marital status, hospital type (small community, larger community and large tertiary care center), location of residence (skilled nursing facility (SNF) vs non- SNF) and insurance status(Commercial, Medicaid, Medicare)), and 2-clinical/anthropomorphic (Charlson score, presence of permanent catheter (permcath) for dialysis, dialysis relevant laboratory values that are validated prognostic markers (minimum albumin within 90 days around index ED visit, lowest and highest serum phosphorus within 900 days around index ED visit, minimum hemoglobin (Hgb) within 900 days around index ED visit, minimum body mass index (BMI) within 900 days around index ED visit, history of diabetes mellitus (which we pulled by searching for Hgba1c>6.0% or an ICD diagnosis of diabetes mellitus within one year prior to index ED visit), and history of heart failure (defined as any ICD diagnosis associated with heart failure within one year prior to index ED visit).

Insurance status was determined based on the “line of business” category defined by claims data captured by “Looking Glass”. Mean socio-economic status (SES) was based on census bureau attributes from census track and census block creating a smaller unit of geographic area, and standardized against the New York State mean score. Each unit represents a multiple of standard deviation, with negative scores representing values below New York State’s mean income.[22] Use of a “permcath” was derived using a data pull instrument from Looking Glass with the words “hemocath”, “permcath” or “tunneled catheter” utilized as identifiers in notes or orders written on the patients at any point during the index ED visit and spanning 30 days prior. The Charlson score was summed by Looking Glass based on an established number of comorbidities.[23] The occurrence of at least one outpatient office visit (not the dialysis facility) within 90 days prior to index ED visit was also captured.

In the final model, data was missing for <12.5% of the data points. The following variables had the most missing data points (in decreasing order): BMI(193 missing), Phosphorus(186 missing), SES (149 missing), and Hemoglobin and Phosphorus (50 missing in each category). We did sensitivity analysis to examine the association of each variable with outcome in the final model using only the cohort with missing data in each variable and did not find any change in the significant associations with outcome.

### Variable transformation

The race category was reclassified after including Ethnicity data. Four categories were devised including: 1-non-hispanic White, 2-non-hispanic Black, 3-Hispanic and 4-other. The Marital status category was dichotomized into married or not married, with not married representing single, widowed, and divorced subjects. Insurance status was transformed into 3 main categories based on primary insurance coverage: 1) Medicare, 2)Medicaid, 3)Commercial. We dichotomized the age variables into those older than 44 years and those 44 years or younger based on the cutoff provided by the USRDS, after finding a significant interaction between gender and age with respect to number of avoidable ED visits.[1] We also dichotomized SES around the New York State mean level (SES = 0).

### Statistical analysis

Differences in demographics and clinical variables based on sex were examined using T-tests or Wilcoxon rank-sum tests for continuous variables and chi-square or Fisher’s exact tests for categorical variables. The variables were divided into demographic, socioeconomic and clinical/anthropomorphic categories. We did bivariate testing on the following variables:1)socio-economic: marital status, SES, residence (SNF vs not), hospital catchment area, and insurance status and 2)clinical: Charlson score, perm-catheter as dialysis access, albumin, minimum hemoglobin around 900 days of index ED visit, maximum phosphorus within 900 days around index ED visit, minimum BMI within 900 days around index ED visit, history of DM, history of heart failure and number of outpatient office visits within 90 days prior to index ED visit. Negative binomial regression was used to examine the association of total ED visits with each individual variable. Multivariate models were then built using the exposure variable: sex, and adjusting for race and age. Because sex, age and race are ancestors of the other variables in a causal diagram, it was more important to test the other (descendant) variables to see if they acted as confounders or modifiers in the association of gender and outcome. The effects of significant(on bivariate analysis(Table 1)) socio-economic variables were examined (marital status, socio-economic status(SES), and insurance status) on the degree of change in the IRR for sex when added to the model, while adjusting for race and age. We did the same for significant clinical/anthropomorphic variables.(based on Table 1) We used a 25% change in the IRR for sex as the threshold for potential mediation.

**Table 1:** Bivariate associations of variables with sex.

	Female(2019)(43.2%)	Male(2654)(56.8%)	p value
Outcome			
Avoidable ED visits(median(25–75% IQR))	**4(2–9)**	**4(2–7)**	**0.003**
**Demographic Variables**			
Age(mean years +/- SD)	**62.6(15.8)**	**60.7(14.3)**	**<0.001**
Race(n = 4673)(%)			**<0.001**
Non-hispanic White(343)	**120(5.9)**	**223(8.4)**	
Non-hispanic Black(2164)	**1082(53.6)**	**1082(40.8)**	
Hispanic(1789)	**696(34.5)**	**1093(41.2)**	
Other(377)	**121(6.0)**	**256(9.7)**	
Primary Language(%)			**0.005**
English (3747)(80.2))	**1657(82.1)**	**1090(78.7)**	
Not English (926)(19.8)	**362(17.9)**	**564(21.2)**	
**Socio-Economic Variables**			
SES (below state mean) (Median 25–75% IQR) N = 4524	**-2.74(-6.40-(-1.13))**	**-2.59(-5.93-(-1.07)**	**0.03**
SES dichotomized at 0 (state mean)(%)(n = 4524)			**0.014**
< = 0	**1830(90.6)**	**2346(88.4)**	
>0	**189 (9.4)**	**(11.6)**	
Married	**470(23.3)**	**1131(42.6)**	**<0.001**
Not married	**1549(76.7)**	**1523(57.4)**	
Hospital Catchment area(number)(%)			0.89
Tertiary care center (2433(52.1))	1039(51.5)	1394(52.5)	
Medium sized community(1546(33.1))	673(33.3)	873(32.9)	
Small community(611(13.1))	270(13.4)	341(12.8)	
Stand-alone ED(83(1.8))	37(1.83)	46(1.7)	
From SNF(435(10.0%))	178(9.4)	257(10.4)	0.24
Not from SNF (3924(90.0%))	1719(90.6)	2205(89.6)	
Insurance type(%)			**0.016**
Commercial(601(13.9))	**229(12.2)**	**372(15.2)**	
Medicaid(1655(38.3))	**736(39.1)**	**919(37.6)**	
Medicare(2067(47.8))	**915(48.7)**	**1152(47.2)**	
**Clinical/Anthropomorphic**			
BMI(minimum within 900 days around index ED visit)(+/- SD)(n = 4480)	**24.6(8.4)**	**23.8(11.6)**	**0.008**
Min phosphorus (within 900 days)(mg/dL)(+/-SD)(N = 4487)	2.84(1.24)	2.87(1.31)	0.42
Max phosphorus(within 900 days)(mg/dL)(+/-SD)	**7.18(2.37)**	**7.49(2.54)**	**<0.001**
Min albumin (within 90 days)(mg/dL)(+/-SD)(n = 3607)	3.73(0.58)	3.76(0.61)	0.17
Max Hemoglobin (within 900 days) (mg/dL) (= /-SD)(n = 4623)	**12.48(1.65)**	**12.82(1.88)**	**<0.001**
Min Hemoglobin(within 900 days)(mg/dL)(+/-SD)(n = 4623)	**7.65(1.90)**	**7.77(2.0)**	**0.05**
Permanent catheter(%)			0.34
Yes(1033)(22.1%)	433(21.4)	600(22.6)	
No (3640)(77.9%)	1586(78.6)	2054(77.4)	
History of DM(%)			0.14
Yes	1168(57.8)	1478(55.7)	
No	851(42.2)	1176(44.3)	
History of Heart Failure(%)			0.97
Yes	643(31.8)	844(31.8)	
No	1376(68.2)	1810(68.2)	
Charlson score (median) (25–75% IQR)(n = 4673)	5(2–7)	5(2–7)	0.29
Having had at least one office visit within 180 days before index ED visit (%)	118(5.8)	168(6.3)	0.49

ED = emergency department,SES = socio-economic class, SNF = skilled nursing facility, BMI = body mass index, DM = diabetes mellitus

## Results

### Descriptive

Four thousand six hundred and seventy-three subjects on hemodialysis were identified as having at least one avoidable ED visit in the period of 2012–2017 at one of four ED sites affiliated with Montefiore Medical Center, in the Bronx. The mean age for the cohort was 61.6 years(+/-15.0). Two thousand and nineteen (43.2%) were female, 2,164(46.3%) were black, 1789(38.2%) were Hispanic. Nine hundred and twenty six subjects(19.8%) were non-English speaking. One thousand, six hundred and one subjects (34.3%) were married. By definition, all subjects had at least one ED visit during the study period, the median number of avoidable ED visits made by the study subjects over 5 years of observation was 4 (25–75% IQR: 2–8). The maximum number of avoidable ED visits made by any subject was 123.

Female patients on hemodialysis in our Bronx cohort, had a higher number of avoidable ED visits, were older, more commonly black, had lower SES scores and were less commonly married, than their male counterparts.(Table 1) Females less commonly had Commercial insurance, indicating that they were less commonly employed, than males. Furthermore female patients on hemodialysis had higher BMIs, lower maximum phosphorus levels and lower maximum and minimum hemoglobin levels within 900 days around the time of the index ED visit, than males.(Table 1)

In analysis of the association of individual variables with total number of avoidable ED visits Black race, lower SES status (dichotomized at New York State Mean), non-commercial insurance, having a higher maximum phosphorus level, a lower minimum Hgb and higher maximum Hgb within 900 days around the index ED visit were all associated with a higher number of avoidable ED visit, while female sex was not (Table 2) Being married, having higher minimum BMI, higher minimum Hemoglobin (within 900 days around index ED visit) were associated with a lower number of avoidable ED visits per patients per year (Table 2).

**Table 2:** Univariate analysis of the association of variables with total ED visits.

Variables	Unadjusted IRR N = 4673	95% C.I.
Age (for every year)	1.00	1.00–1.00
Sex		0.99–1.12
Male	1.0	
Female	1.05	
Race		
White	**1**	
Black	**1.14**	**1.01–1.29**
Hispanic	**1.12**	**0.99–1.27**
Other	**0.90**	**0.77–1.06**
Language		0.98–1.14
English	1	
Not-English	1.06	
SES dichotomized at 0		0.69–0.85
<0	1	
> = 0	**0.77**	
Living in a skilled nursing facility(n = 4358)		0.99–1.24
No	1	
Yes	1.11	
Married		0.88–0.99
No	1.0	
Yes	**0.93**	
Insurance type		
Commercial	1	
Medicaid	**1.25**	1.14–1.36
Medicare	**1.28**	1.17–1.39
Minimal BMI (for every one unit increase) (n = 4,480)	**0.98**	0.98–0.98
Maximum Phosphorus (for every 1mg/dL increase)(n = 4487)	**1.12**	1.11–1.13
Maximum Hemoglobin (for every 1mg/dL increase in Hgb)(n = 4623)	**1.06**	1.04–1.07
Minimum Hemoglobin (for every 1mg/dL increase)(n = 4623)	**0.85**	0.84–0.87

IRR = incident rate ratio, SES = socio-economic class

In unadjusted analysis the association of sex and avoidable ED visit rate did not reach statistical significant. (Table 2) Marital status acted as a potential mediator in the association of sex and outcome (number of ED visits)(coefficient for sex changed from 0.051 to 0.039 when marital status was added) as did SES dichotomized around New York State Mean (0.051 to 0.043), minimum Hgb (coefficient for gender changed from 0.051 to 0.074 when minimum hemoglobin was added) and minimum BMI (0.051–0.031).(Table 3) Testing for interaction between sex and marital status, sex and SES, sex and insurance status showed non-significant p values in the final model.(p for interaction = 0.28, 0.85, 0.53, respectively). However, testing for interactions between sex and race category, and sex and age with respect to outcome resulted in significant p values. (p = 0.041 for gender and race; p = 0.024 for gender and age). Younger females had a significantly higher rate of ED visits as compared to older females. (IRR 1.03 for those female subjects older than 44 years old, vs 1.17 for those females subjects younger than 44 years old.)

**Table 3:** Percent change in the coefficient for gender with the addition of each variable in the model.

Variable *Socioeconomic*	Percent change in coefficient (IRR for sex without new variable, IRR with new variable added to model)
Marital status (married vs not)	-26.9%(1.053, 1.039)
SES (dichotomized at 0)	-17.3%(1.053, 1.044)
Insurance status	+13.4%(1.053, 1.066)
*Clinical/Anthropomorphic*	
Minimum BMI	+36.5%(1.053, 1.074)
Minimum Phosphorus	-13.5%(1.053, 1.047)
Maximum Hemoglobin	+25%(1.053, 1.068)
Minimum Hemoglobin	-40.4%(1.053, 1.032)

SES = socio-economic status, BMI = body mass index.

We built a multivariate model with number of avoidable ED visits as our outcome and stratified our analysis based on race categories.(Table 4) The association of female sex and avoidable ED visits was only significant in the non-hispanic White population, and not the other race groups.(Table 4) Non-commercially insured patients were more likely to have more ED visits. Marital status was a possible mediator in Hispanics, with lower risk among married patients. (Table 4)

**Table 4:** Multivariate analysis of the association of gender and outcome stratified by race categories.

Variables	Adjusted IRR in non-hispanic Whites(95% CI) N = 343	Adjusted IRR in non-hispanic Blacks(95% CI) N = 2162	Adjusted IRR In hispanics (95% CI) N = 1789	Adjusted IRR in “other” races (95% CI) N = 377
Gender				
Male	1	1	1	1
Female	**1.30(1.01–1.69)**	1.04(0.96–1.13)	1.05(0.96–1.15)	1.19(0.99–1.42)
Age (for every 1 year increase)	1.00(0.99-.1.01)	1.00(1.00–1.00)	**1.01(1.00–1.01)**	**1.01(1.00–1.02)**
SES dichotomized				
<0	1	1	**1**	1
> = 0	0.84(0.64–1.11)	**0.75(0.65–0.89)**	**0.96(0.82–1.14)**	0.61(0.46–0.81)
Marital status				
Not married	1	1	1	1
Married	1.00(0.78–1.29)	0.99(0.90–1.08)	0.90(0.82–0.99)	0.98(0.82–1.17)
Insurance status				
Commercial	**1**	**1**	1	1
Medicaid	**1.52(1.03–2.26)**	**1.33(1.18–1.51)**	1.13(0.98–1.30)	0.96(0.75–1.22)
Medicare	**1.48(1.01–2.17)**	**1.17(1.03–1.33)**	1.15(0.99–1.34)	0.92(0.71–1.20)
BMI (minimum) within 90 days around index ED visit (for every one unit increase)	**0.99(0.97–1.00)**	**0.99(0.98–0.99)**	**1.00(0.99–1.00)**	**0.98(0.97–0.99)**
Hemoglobin (minimum) within 900 days around index ED visit (for every 1gm/dL increase)	**0.90(0.82–0.98)**	**0.90(0.87–0.92)**	**0.87(0.85–0.90)**	**0.87(0.84–0.93)**
Hemoglobin (maximum) within 900 days around index ED visit (for every 1gm/dL increase)	1.03(0.95–1.11)	1.09(1.06–1.10)	**1.12(1.09–1.15)**	**1.10(1.05–1.16)**
Phosphorus (maximum) within 90 days around index ED visit (for every 1gm/dL increase)	**1.11(1.05–1.17)**	**1.08(1.06–1.10)**	**1.08(1.06–1.11)**	**1.10(1.05–1.14)**

IRR = incident rate ratio, CI = confidence interval, ED = emergency department.

## Discussion

Our analysis in a cohort of patients on hemodialysis living in the Bronx, shows that the association of sex with avoidable ED visit outcomes is not significant and varies by race, with significance only in non-hispanic Whites, and in younger patients. Furthermore low SES, anemia, high phosphorus, and low Hgb potentially mediate the association of sex with risk of preventable ED visits. Females were poorer; less commonly had commercial insurance and were less commonly married than their male counterpart. SES and family support have been shown to be associated with dialysis adherence, mental well-being and hard outcomes in this population.[24, 25] The association of female sex and inpatient resource utilization is shown in multiple studies that examine databases from national cohort of patients on hemodialysis, and is consistent across multiple strata of age and race.[11, 12, 15, 16, 19, 26] These studies of national cohorts, however, do not adjust for regional/community level determinants of health (race, access to care, psychosocial factors and health literacy) which have been shown to be important in the outcomes of the ESRD population.[8, 9, 27–29] Regional and community level disparities with regard to access to care and psychosocial stressors contribute to hospitalization outcomes.[10] In communities with low SES and Black majority, patients on hemodialysis are at a higher risk for mortality as compared to White communities and patients with higher SES.[8, 28] Differences in socio-economic status and social support also play a role in adherence behaviors and high frequency of ED visits.[25, 28, 30–32] Thus differences in ED utilization between sexes could be partially driven by differences in access to care, SES, social support and dialysis related clinical status. [33–35] The association of “avoidable” hospital ED utilization with female sex may be in part mediated by the lack of social support and economic disadvantage in the Bronx population. Our results are dissimilar to the USRDS data and the data from Adams et al. highlighting the need to validate these findings within different communities in order to tailor interventions.[9, 10]

Our results also show that Medicaid primary insurance and lower than the NYS mean SES were both significantly associated with rate of avoidable ED visits, supporting the notion that access to care is partially dependent on financial resources and mediated by insurance status.[10, 14] Patients with Medicaid insurance have a higher risk of ED visits in the general population and this is attributable to lack of access to adequate outpatient care and overutilization of safety net hospitals for routine care in the underserved communities.[36] This is also relevant to the dialysis population because access to nephrology care, transplant services and primary care is associated with hard outcomes.[8, 28] Ideally, a combination of databases that account for community/neighborhood based determinants of health and clinical parameters can help elucidate the role of sex (and associated demographic characteristics) with hospitalization outcomes. Because of these nuances in the association of sex and ED visit outcomes, one must consider local case-mix and community level factors when applying national benchmarks to outcomes in these populations.

Our findings support the notion that different social, economic and clinical drivers apply to different race/ethnic and age groups with regard to avoidable ED visits and that setting quality benchmarks based on national data that do not account for these differences may be harmful as far as reimbursement decisions. A recent study conducted by DOPPS showed that women’s survival advantage (in the general population) was markedly diminished in hemodialysis patients. [37] Fewer female patients were on hemodialysis than males and females had a higher rate of catheter use in the international cohort.[37] The reason for the lack of survival benefit in women on dialysis cannot be inferred, however, and may be related to the high mortality risk in patients on hemodialysis, in general. Our study differs from similar studies that examine associations with hospital resource utilization in several important ways. 1) our outcome variable was ED visits not associated with an inpatient admission which we utilized in an effort to identify a pattern of ED use that is potentially avoidable, 2) our patient population was defined by having made at least one visit to the ED (an already higher risk group) within 5 years of observation, 3) our study population was relatively homogenous with respect to low SES and racial makeup, limiting generalizability. Notwithstanding these limitations our study has the advantage of examining the relevance of nationally defined risk factors of hospital resource utilization in a mostly minority and economically vulnerable patient population. Accordingly, the association of sex with ED visits is significant for White patients only, while Medicaid and SES had a significant association with outcomes in Whites and Blacks. We showed that low BMI, low hemoglobin and high phosphorus, clinical variables that represent inflammation/malnutrition, were associated with avoidable ED visits across all race and age groups. This latter has been described by multiple studies and is not unique to our population.[38–40]

Our analysis has several important limitations. The sample of patients is drawn from a single center’s database and created based on having had at least one ED visit. This not only limits the generalizability of the findings but also limits the analysis because of a narrow range of SES and racial makeup of the study population. We believe that our cohort is a fair representation of all of the patients on hemodialysis in the area because of the range of time used to create the cohort and the frequency with which patients on hemodialysis make ED visits. Even though our center is the predominant center in the Bronx and the hospital fidelity in our dialysis population is quite high, there is a possibility that some ED visits were made to two smaller community hospitals in the Bronx not included in the study. Both of these limitations may present a non-differential sampling bias, as there is no reason to believe that the sampling is different for males vs females based on our criteria. It is possible that in low SES communities’ lack of social support has a differential effect on sexes in so far as young women have the added burden of childcare and financial support for families contributing to the burden of their daily lives.[24, 25, 41, 42] Dialysis vintage and dietary and dialysis treatment adherence were also missing from the analysis. Again there is little reason to believe that these variables would be a source of differential bias based on gender, however we do acknowledge that they are important areas to explore in an effort to further refine our findings. Furthermore dialysis treatment adherence was partially represented by phosphorus and hemoglobin levels.

## Conclusion

In conclusion, the association of female sex with avoidable ED visits was not significant in a lower income, mostly minority, ESRD patient population, unlike what is described in national cohorts. Potential mediators of the significant association of female sex and ED visits in other populations, and our own subpopulation, include marital status, SES and degree of anemia. A greater appreciation of community level determinants of ED use is needed to tailor successful interventions that mitigate hospital resource utilization in the ESRD population.

## Supporting information

S1 Data De-identified database. (DTA)
